# Supplementary material for: Insights into Muscle Contraction Derived from the Effects of Small-Molecular Actomyosin-Modulating Compounds
Source: Int J Mol Sci. 2022 Oct 11;23(20):12084. doi: 10.3390/ijms232012084 (PMC9603234; doi:10.3390/ijms232012084)
Supplement: Supplementary file 1 [file ijms-23-12084-s001.zip › ijms-1925306-supplementary.pdf]

International Journal of
Molecular Sciences

MDPI

Supplementary Information

Insights into Muscle Contraction Derived from the Effects of Small-Molecular Actomyosin-Modulating Compounds

Alf Månsson [1,*] and Dilson E. Rassier [2]

1 Department of Chemistry and Biomedical Sciences, Linnaeus University, SE391 82 Kalmar, Sweden
2 Department of Kinesiology and Physical Education, McGill University, Montreal, QC H2W 1S4, Canada; e-mail@e-mail.com
* Correspondence: alf.mansson@lnu.se; Tel.: +46-708-866243

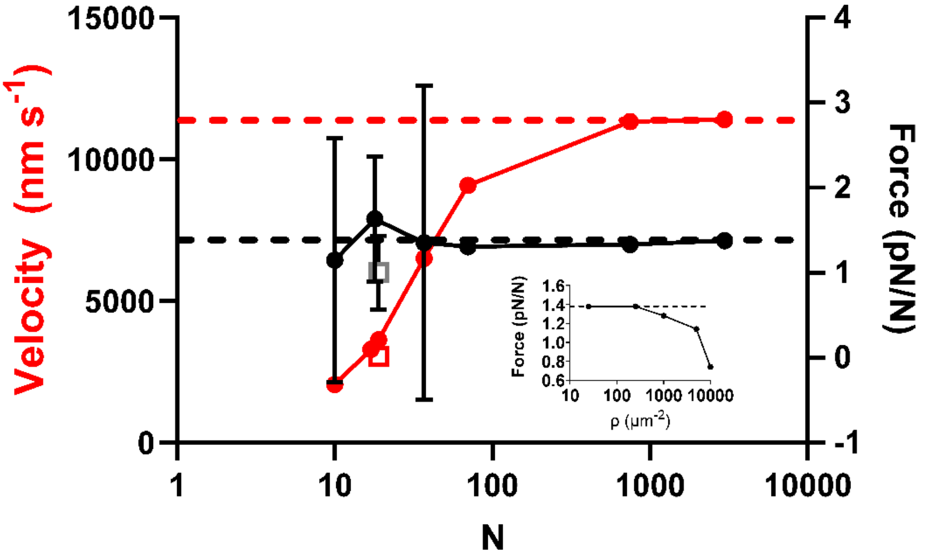

Figure S1. Monte Carlo simulation-based predictions for force and velocity vs. the number of available myosin heads (N). The simulations (filled symbols) assume a myosin head density on the surface of 25 µm^{-2} and a width of the band with available heads around the filament of 30 nm. The variation in N was achieved by varying the assumed filament length. The case N=19 was also achieved by assuming the combination of myosin head density on the surface of 200 µm^{-2} and filament length of 3.1 µm (open square). Inset: Force per available myosin heads as a function of the assumed surface density of myosin heads in the simulations when N is kept constant at approximately 3000 by simultaneous variation in the filament length, e.g., for ρ=100 µm^{-2}, L=1000 µm, whereas for ρ=10000 µm^{-2}, L=10 µm.

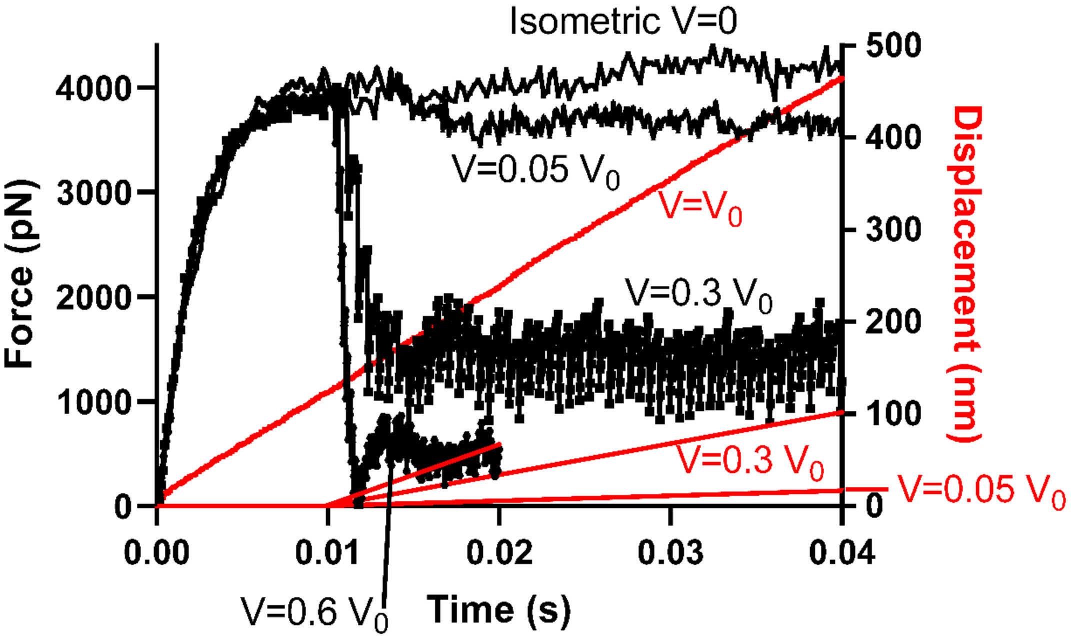

Figure S2. Monte Carlo simulation of force–velocity data for large number of myosin heads (N, 2975-2990). The simulations assume a myosin head density on the surface of 25 μm^{-2} and a filament length of 4000 μm; see Methods for more details. Different modes of simulation (isometric with ramp shortening and force-clamped to 0 pN) to simulate force–velocity data for velocities from V=0 nm/s – V_0 are also described in the Methods. The large regular variability in force during ramp shortening, particularly for V=0.3V_0, is due to the approximation of the shortening ramp by consecutive shortening steps of approximately 2 nm each imposed every 0.6 ms.

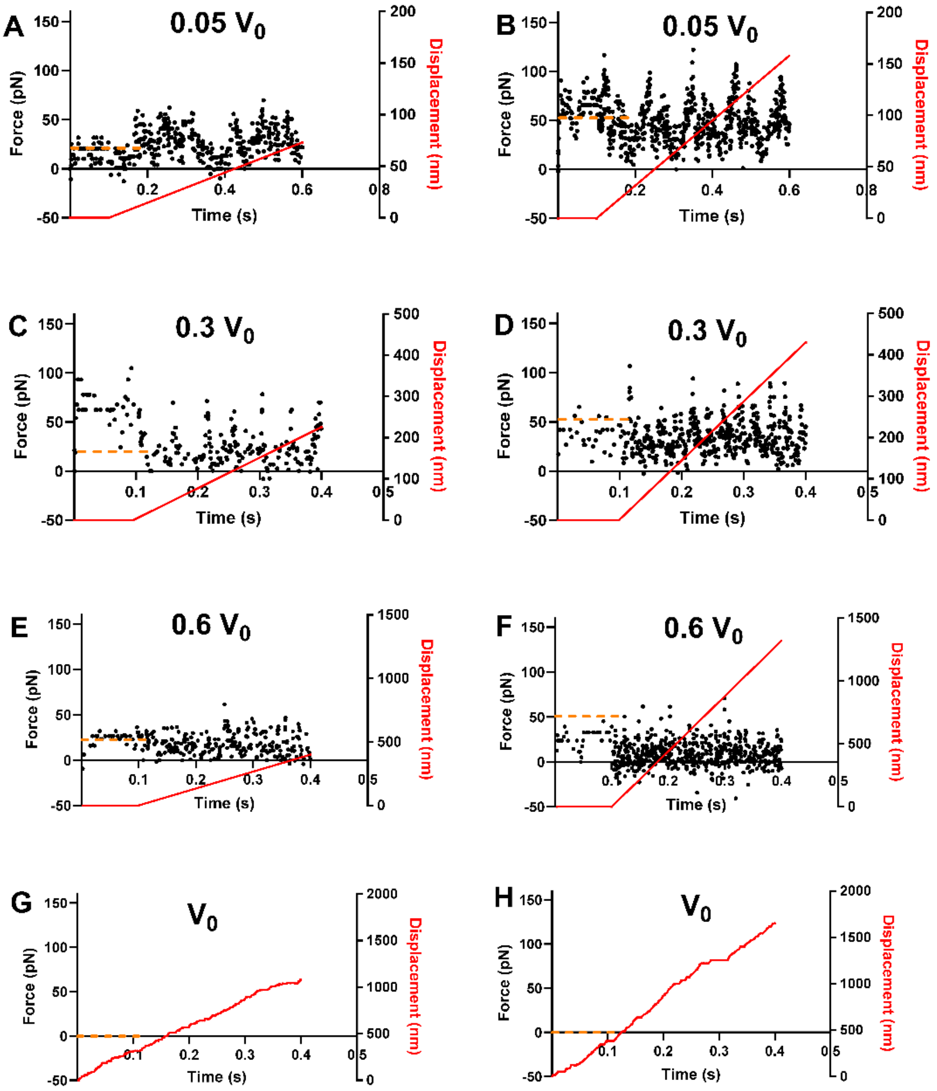

Figure S3. Monte Carlo simulation of FV data for N=17-19 (left panels) and N=34-39 (right panels). In **A-F,** shortening ramps (displacements, right axis, red) were imposed when steady-state isometric force had been attained with velocities of V_0 varying between 5 % and 60 %. **G-H**. The displacements required to keep force constant at zero, corresponding to shortening at maximum velocity, V_0. Note the appreciable variability in force (black symbols) between both runs (particularly isometric force) and within runs (particularly force during shortening). The variability in isometric force between runs is clear by comparing the simulated data to the average isometric force obtained in all runs (orange dashed line).

Basis for in-between runs variability in Monte Carlo simulations of isometric force at low N

For the total number of myosin heads, N, available along a given actin filament, the number (N_{att}) that will be in the range with appreciable probability of attachment (approximately 5 nm) will be binomially distributed (Bin (N,p)) where p = 5/36. This follows because the initial random assignment of myosin heads relative to the nearest actin sites (bins along x-axis) will either be in the attachment range (with probability p=5/36≈0.139) or not (1-p). Moreover, the assignment of subsequent heads to different x values is approximately independent of previous assignments due to the great excess in the number of actin sites (N_{act}) over the number of myosin heads.
